# Association of human papillomavirus 16 E6 variants with cervical carcinoma and precursor lesions in women from Southern Mexico

**DOI:** 10.1186/s12985-015-0242-3

**Published:** 2015-02-22

**Authors:** Julio Ortiz-Ortiz, Luz del Carmen Alarcón-Romero, Marco Antonio Jiménez-López, Víctor Hugo Garzón-Barrientos, Itzel Calleja-Macías, Hugo Alberto Barrera-Saldaña, Marco Antonio Leyva-Vázquez, Berenice Illades-Aguiar

**Affiliations:** Laboratorio de Biomedicina Molecular, Unidad Académica de Ciencias Químico Biológicas, Universidad Autónoma de Guerrero, Chilpancingo, Guerrero México; Laboratorio de Citopatología, Unidad Académica de Ciencias Químico Biológicas, Universidad Autónoma de Guerrero, Chilpancingo, Guerrero México; Instituto Estatal de Cancerología “Dr. Arturo Beltrán Ortega”, Acapulco, Guerrero México; Molecular Biology and Biochemistry, School of Biological Sciences, University of California Irvine, Irvine, CA USA; Departamento de Bioquímica y Medicina Molecular, Facultad de Medicina, Universidad Autónoma de Nuevo León, Monterrey, Nuevo León México

**Keywords:** HPV 16 variants, Cervical cancer, Precursor lesions

## Abstract

**Background:**

HPV 16 is the cause of cervical carcinoma, but only a small fraction of women with HPV infection progress to this pathology. Besides persistent infection and HPV integration, several studies have suggested that HPV intratype variants may contribute to the development of cancer. The purpose of this study was to investigate the nucleotide variability and phylogenetically classify HPV 16 E6 variants circulating over a period of 16 years in women from Southern Mexico, and to analyze its association with precursor lesions and cervical carcinoma.

**Methods:**

This study was conducted in 330 cervical DNA samples with HPV 16 from women who were residents of the State of Guerrero, located in Southern Mexico. According of cytological and/or histological diagnosis, samples were divided into the following four groups: no intraepithelial lesion (n = 97), low-grade squamous intraepithelial lesion (n = 123), high-grade squamous intraepithelial lesion (n = 19) and cervical carcinoma (n = 91). HPV 16 E6 gene was amplified, sequenced and aligned with reference sequence (HPV 16R) and a phylogenetic tree was constructed to identify and classify HPV 16 variants. Chi squared was used and data analysis and statistics were done with SPSS Statistics and STATA softwares.

**Results:**

Twenty seven HPV 16 E6 variants were detected in women from Southern Mexico, 82.12% belonged to the EUR, 17.58% to AA1 and 0.3% to Afr2a sublineages. The most common was E-G350 (40%), followed by E-prototype (13.03%), E-C188/G350 (11.82%), AA-a (10.61%), AA-c (6.07%) and E-A176/G350 (5.15%). Eight new E6 variants were found and 2 of them lead to amino acid change: E-C183/G350 (I27T) and E-C306/G350 (K68T). The HPV 16 variant that showed the greatest risk of leading to the development of CC was AA-a (OR = 69.01, CI = 7.57-628.96), followed by E-A176/G350 (OR = 39.82, CI = 4.11-386.04), AA-c (OR = 21.16, CI 2.59-172.56), E-G350 (OR = 13.25, CI = 2.02-87.12) and E-C188/G350 (OR = 10.48, CI = 1.39-78.92).

**Conclusions:**

The variants more frequently found in women with cervical carcinoma are E-G350, AA-a, AA-c, E-C188/G350 and E-A176/G350. All of them are associated with the development of cervical carcinoma, however, AA-a showed the highest association. This study reinforces the proposal that HPV 16 AA-a is an oncogenic risk for cervical carcinoma progression in Mexico.

**Electronic supplementary material:**

The online version of this article (doi:10.1186/s12985-015-0242-3) contains supplementary material, which is available to authorized users.

## Background

Infection by high risk human papillomavirus (HR-HPV) is necessary for the development of cervical carcinoma (CC) [1] and HPV 16 is the cause of more than half of CC worldwide [2]. Only a small fraction of women with HPV infection may progress to cervical carcinoma; however, the factors that favor this progression are still poorly understood. Besides persistent infection and HPV integration [3,4], several studies have suggested that HPV intratype variants may contribute to cancer development [4-7].

Like other HR-HPV, HPV 16 has well preserved distinctive intratypic variants by geographical origin [8], their global distribution and risk of cervical carcinoma appears to be dependent on the population [9,10]. Its phylogeny reflects evolutionary divergence associated with human migration patterns, suggesting that they may have co-diversified as human populations expanded worldwide [11]. HPV 16 variants have been classified into 4 major lineages and 9 sublineages based on common LCR and E6 single polymorphisms: (1) European-Asian (EAS), including European (EUR) and Asian (As) sublineages; (2) African 1 (AFR1), including Afr1a and Afr1b sublineages; (3) African 2 (AFR2), including Afr2a and Afr2b sublineages; and (4) Asian American/North American (AA/NA), including Asian American 1 (AA1), Asian American 2 (AA2) and North American (NA) sublineages [12].

Several reports have shown the presence of common polymorphisms that generate amino acid changes in the E6 oncoprotein, one of them is T350G, and is present in the four lineages. T350G causes a leucine to valine change (L83V), that leads to the split of the EUR sublineage into three classes, 350 T (prototype sequence), 350C and 350G. Other polymorphisms including A131G, G132C, C143G, G145T, G176A, T178G and C335T generate the amino acid changes R10G/I, Q14H/D, D25E/N, I27R and H78Y, respectively [13]. It has been suggested that these polymorphisms and the subsequent amino acid changes in E6 HPV 16 variants may influence the persistence of HPV infection and its progression to cervical carcinoma [4,14-18].

Epidemiologic data shows that regions with high incidence of cervical carcinoma like Latin America, Africa and Asia, also have a high prevalence of sublineages AA and Af [9]. Studies in Mexico have reported that persistent infection and risk of progression to cervical carcinoma is higher when HPV infection is caused by AA sub-lineages compared with EUR sublineages [5,19-21].

Social disparities like access to social security health care services, ethnic groups, residence and socioeconomic level are factors associated with cervical carcinoma development [22]. The State of Guerrero, located in Southern Mexico, is the second poorest state in Mexico and a majority of inhabitants have a very low socioeconomic level. In this region, cervical carcinoma is the most common type of cancer in women and has the fourth highest mortality rate in the country with 12.5 deaths per 100,000 women, compared to the national mortality rate of 9.1 per 100,000 in 2008 [23].

We have previously shown that HPV 16 was the most commonly identified HPV genotype in cervical carcinoma and high grade squamous intraepithelial lesions in women from the State of Guerrero. We studied a sample of HPV 16 positive women and found E and AA variants [24]. It has been proposed that variants AA of HPV 16 are more oncogenic than E variants [5,25].

Knowing the regional variants of HPV 16 is of great value for evolutionary, phylogenetic, epidemiological and biological analysis [13]. To further analyze the regional variants of HPV 16, the aim of this study was to investigate the nucleotide variability and phylogenetically classify HPV 16 E6 variants circulating over a period of 16 years in the Southern Mexican population, and to analyze its association with the whole spectrum of disease from no intraepithelial lesion in cervical epithelium to cervical carcinoma.

The most dominant HPV variants were detected in low and high-grade squamous intraepithelial lesion, cervical carcinoma and no intraepithelial lesion, and 8 novel HPV 16 variants were found. An association between E-G350, E-A176/G350, E-C188/G350, AA-a and AA-c variants and the risk of developing cervical carcinoma was shown in this study.

## Results

### HPV 16 E6 variants and phylogenetic analysis

The variant analysis for the E6 gene was carried out in 330 HPV16 samples from all study groups. Using the HPV 16 R (Los Alamos National Laboratory, http://www.ncbi.nlm.nih.gov/nuccore/NC_001526.2) as a reference sequence, a total of 27 variants were detected, 8 of them were new. Sequence analysis showed substitution in 29 nucleotides located between positions 104 and 559 in the E6 sequence with a predicted amino acid change (Table 1).

**Table 1:** **Phylogenetic classification, nucleotide sequence variations and frequency of HPV-16 E6 variants identified in women from Southern Mexico**

**Variants**	**HPV 16 E6 nucleotide position**																										**Predicted amino acid change E6 protein (151 aa form)**	**Frequency**	
	**109**	**110**	**131**	**132**	**143**	**145**	**176**	**182**	**183**	**185**	**188**	**189**	**256**	**257**	**271**	**286**	**289**	**306**	**310**	**335**	**350**	**403**	**424**	**442**	**532**	**535**		**N=330**	**%**
HPV 16R	T	C	A	G	C	G	G	A	T	T	G	A	C	A	T	T	A	A	C	C	T	A	A	A	A	A			
Lineage EAS/Sublineage EUR																													
Class E-T350																												**51**	**15.45%**
E-Prototype	-	-	-	-	-	-	-	-	-	-	-	-	-	-	-	-	-	-	-	-	-	-	-	-	-	-	No change	43	13.03%
E-G131	-	-	G	-	-	-	-	-	-	-	-	-	-	-	-	-	-	-	-	-	-	-	-	-	-	-	R10G	8	2.42%
Class E-G350																												**220**	**66.67%**
E-G350	-	-	-	-	-	-	-	-	-	-	-	-	-	-	-	-	-	-	-	-	G	-	-	-	-	-	L83V	132	40.00%
E-C109/G350	c	-	-	-	-	-	-	-	-	-	-	-	-	-	-	-	-	-	-	-	G	-	-	-	-	-	L83V	6	1.82%
E-G110/G350	-	G	-	-	-	-	-	-	-	-	-	-	-	-	-	-	-	-	-	-	G	-	-	-	-	-	Q3E/L83V	2	0.61%
E-G131/G350	-	-	G	-	-	-	-	-	-	-	-	-	-	-	-	-	-	-	-	-	G	-	-	-	-	-	R10G/L83V	2	0.61%
E-G131/C188/G350	-	-	G	-	-	-	-	-	-	-	C	-	-	-	-	-	-	-	-	-	G	-	-	-	-	-	R10G/E29Q/L83V	2	0.61%
E-A176/G350	-	-	-	-	-	-	A	-	-	-	-	-	-	-	-	-	-	-	-	-	G	-	-	-	-	-	D25N/L83V	17	5.15%
**E-A176/G424/G350	-	-	-	-	-	-	A	-	-	-	-	-	-	-	-	-	-	-	-	-	G	-	g	-	-	-	D25N/L83V	1	0.30%
E-T182/G350	-	-	-	-	-	-	-	T	-	-	-	-	-	-	-	-	-	-	-	-	G	-	-	-	-	-	I27L/L83V	4	1.21%
E-C182/G350	-	-	-	-	-	-	-	C	-	-	-	-	-	-	-	-	-	-	-	-	G	-	-	-	-	-	I27L/L83V	2	0.61%
**E-C183/G350	-	-	-	-	-	-	-	-	*C*	-	-	-	-	-	-	-	-	-	-	-	G	-	-	-	-	-	I27T/L83V	3	0.91%
E-G185/G350	-	-	-	-	-	-	-	-	-	G	-	-	-	-	-	-	-	-	-	-	G	-	-	-	-	-	L28V/L83V	2	0.61%
E-A188/G350	-	-	-	-	-	-	-	-	-	-	A	-	-	-	-	-	-	-	-	-	G	-	-	-	-	-	E29K/L83V	1	0.30%
E-C188/G350	-	-	-	-	-	-	-	-	-	-	C	-	-	-	-	-	-	-	-	-	G	-	-	-	-	-	E29Q/L83V	39	11.82%
**E-C188/G310/G350	-	-	-	-	-	-	-	-	-	-	C	-	-	-	-	-	-	-	G	-	G	-	-	-	-	-	E29Q/F69L/L83V	2	0.61%
**E-G189/T256/G350	-	-	-	-	-	-	-	-	-	-	-	G	t	-	-	-	-	-	-	-	G	-	-	-	-	-	E29G/L83V	1	0.30%
E-G257/G350	-	-	-	-	-	-	-	-	-	-	-	-	-	G	-	-	-	-	-	-	G	-	-	-	-	-	I52L/L83V	1	0.30%
**E-C306/G350	-	-	-	-	-	-	-	-	-	-	-	-	-	-	-	-	-	*C*	-	-	G	-	-	-	-	-	K68T/L83V	1	0.30%
E-C442/G350	-	-	-	-	-	-	-	-	-	-	-	-	-	-	-	-	-	-	-	-	G	-	-	C	-	-	L83V/E113D	1	0.30%
**G535/G350	-	-	-	-	-	-	-	-	-	-	-	-	-	-	-	-	-	-	-	-	G	-	-	-	-	*g*	L83V	1	0.30%
Lineage AA/NA/Sublineage AA1																													
Class AA-a																												**36**	**10.91%**
AA-a	-	-	-	-	-	T	-	-	-	-	-	-	-	-	-	a	g	-	-	T	G	-	-	-	g	-	Q14H/H78Y/L83V	35	10.61%
**AA-a/C188	-	-	-	-	-	T	-	-	-	-	C	-	-	-	-	a	g	-	-	T	G	-	-	-	g	-	Q14H/E29Q/H78Y/L83V	1	0.30%
Class AA-c																												**22**	**6.67%**
AA-c	-	-	-	-	-	T	-	-	G	-	-	-	-	-	-	a	g	-	-	T	G	-	-	-	g	-	Q14H/I27R/H78Y/L83V	20	6.07%
**AA-c/G185	-	-	-	-	-	T	-	-	G	G	-	-	-	-	-	a	a	-	-	T	G	-	-	-	g	-	Q14H/I27R/L28V/H78Y/L83V	1	0.30%
AA-c/C271	-	-	-	-	-	T	-	-	G	-	-	-	-	-	c	a	g	-	-	T	G	-	-	-	g	-	Q14H/I27R/H78Y/L83V	1	0.30%
Lineage AFR2/Sublineage Afr2a																													
Class Af2-a																												**1**	**0.30%**
Af2-a/C109/G403	c	-	-	T	G	T	-	-	-	-	-	-	-	-	-	a	g	-	-	T	-	g	-	-	-	-	R10I/Q14D/H78Y	1	0.30%

HPV 16 R: reference sequence. Predicted amino acid change: the amino acid numeration was done using the 151 amino acid E6 oncoprotein form as reference. Capital letters indicate polymorphisms that produce amino acid change. Lower-case letters indicate silent mutations. – Indicate no polymorphism. Italic letters indicate new polymorphisms. Italic capital letters indicate amino acid change. **Indicate new variants.

**Figure 1:**
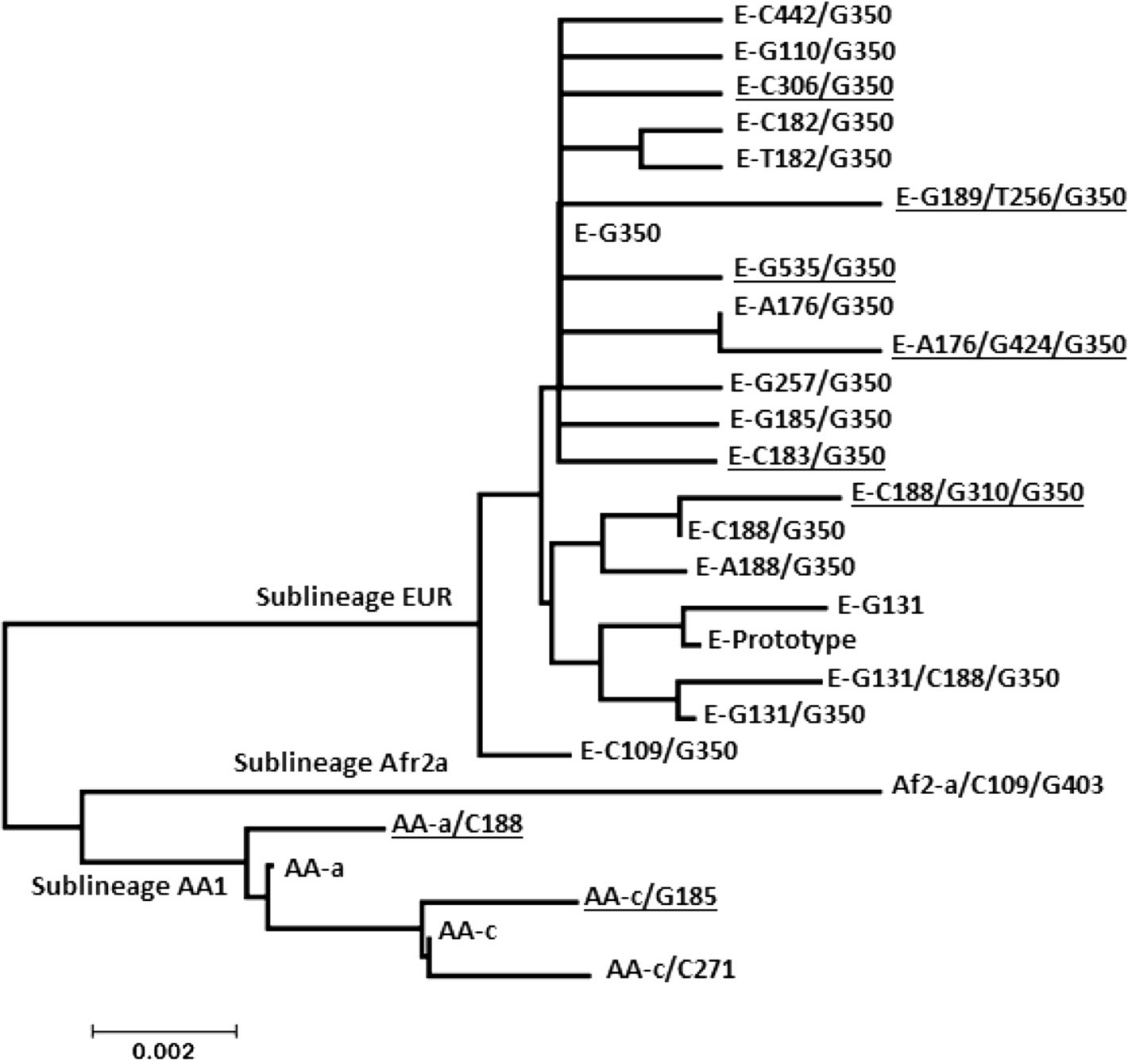
**Phylogenetic tree based in E6 variations of HPV 16.** To classify the newly identified variants, their phylogenetic relationship with previously reported variants were determined, by constructing a phylogenetic tree based on E6 sequences found in Southern Mexico. The new variants are underlined. The tree is drawn to scale with branch lengths measured in the number of substitutions per site. The analysis involved 27 nucleotide sequences. All positions containing gaps and missing data were eliminated. There were a total of 456 positions in the final dataset. Analyses were conducted in MEGA5.2 [43].

The phylogenetic analysis showed that the E6 variants found belong to EUR, AA1 and Afr2a sublineages. Six of the 8 novel variants were related to variants of the EUR sublineages, and 2 to variants of the AA1 sublineage (Figure 1).

The phylogenetic classification of the E6 variants showed that the majority (82.12%) belonged to the EUR sublineage, followed by AA1 (17.58%) and Afr2a (0.3%) sublineages. From sublineage EUR, 15.45% was class E-T350 which includes E-Prototype (13.03%), and 66.67% was class E-G350 finding 19 subclasses. From sublineage AA1, AA-a class was the most frequent (10.91%) followed by AA-c class (6.67%). From the 27 variants found in this study, the most common E6 variant was E-G350 (40%), followed by E-prototype (13.03%), E-C188/G350 (11.82%), AA-a (10.61%), AA-c (6.07%) and E-A176/G350 (5.15%). The 8 novel variants are subclasses, 6 are subclasses of class G-350, one is subclass of class AA-a and the last one is subclass of class AA-c (Table 1). E-C183/G350, E-C306/G350 and E-G535/G350 show polymorphisms that so far have not been reported, E-C183/G350 leads to the amino acid change I27T, E-C306/G350 changes K68T and E-G535/G350 does not lead to any amino acid change (Table 1).

### HPV 16 E6 variants in cervical carcinoma and precursor lesions

A total of 330 samples with HPV16 were analyzed. The histology of the 91 cervical carcinoma identified 76 (83.5%) as squamous cell carcinoma (SCC), 13 (14.3%) as adenocarcinoma (ADC) and 2 (2.2%) as other epithelial tumors. The majority of cases of cervical carcinomas were found in the FIGO stage IIB (34%). Additionally, 19 samples were HSIL, 123 LSIL and 97 with non-IL (11 with inflammation and 86 with normal Pap smears).

HPV 16 AA variants were more common in ADC (46.15%) than in SCC (30.27%). AA-a variants increased their frequency according to the degree of evolution of cervical lesion: 38.46% in ADC, 15.79% in SCC, 15.79% in HSIL, 8.94% in LSIL and 3.09% in non-IL. HPV 16 E variants were the most common in SCC (69.75%), HSIL (78.94%), LSIL (87.79%) and non-IL (89.69%). HPV 16 E-G350 class was the most frequent in all groups, E-Prototype, on the other hand, was not detected in ADC and other epithelial tumors, and only 2.63% of SCC, 5.26% of HSIL, 18.7% of LSIL and 17.53% of non-IL (Table 2). The Af variants were not found in any cervical carcinoma.

**Table 2:** **Distribution of HPV 16 E6 variants for diagnostic category**

**Variant**	**Non-IL N=97**	**LSIL N=123**	**HSIL N=19**	**SCC N=76**	**ADC N=13**	**Other Epithelial Tumor N=2**	**Cervical carcinoma N=91**	**Total N=330**
E variants	**87 (89.69%)**	**108 (87.79%)**	**15 (78.94%)**	**53 (69.75%)**	**7 (53.84%)**	**1 (50.0%)**	**61 (67.05%)**	**271 (82.12%)**
Class E-T350	**23 (23.72%)**	**23 (18.7%)**	**1 (5.26%)**	**4 (5.26%)**	**-**	**-**	**4 (4.4%)**	**51 (15.45%)**
E-Prototype	17 (17.53%)	23 (18.7%)	1 (5.26%)	2 (2.63%)	-	-	2 (2.2%)	43 (13.03%)
E-G131	6 (6.19%)	-	-	2 (2.63%)	-	-	2 (2.2%)	8 (2.42%)
Class E-G350	**64 (65.97%)**	**85 (69.09%)**	**14 (73.68%)**	**49 (64.49%)**	**7 (53.84%)**	**1 (50.0%)**	**57 (62.65%)**	**220 (66.67%)**
E-G350	39 (40.21%)	56 (45.53%)	6 (31.58%)	28 (36. 84%)	3 (23.08%)	-	31 (34.07%)	132 (40.00%)
E-C109/G350	-	3 (2.44%)	1 (5.26%)	2 (2.63%)	-	-	2 (2.2%)	6 (1.82%)
E-G110/G350	1 (1.03%)	1 (0.81%)	-	-	-	-	-	2 (0.61%)
E-G131/G350	1 (1.03%)	-	-	1 (1.32%)	-	-	1 (1.1%)	2 (0.61%)
E-G131/C188/G350	1 (1.03%)	1 (0.81%)	-	-	-	-	-	2 (0.61%)
E-A176/G350	3 (3.09%)	5 (4.07%)	1 (5.26%)	5 (6.58%)	2 (15.38%)	1 (50.0%)	8 (8.79%)	17 (5.15%)
**E-A176/G424/G350	-	-	-	1 (1.32%)	-	-	1 (1.1%)	1 (0.30%)
E-T182/G350	-	1 (0.81%)	1 (5.26%)	2 (2.63%)	-	-	2 (2.2%)	4 (1.21%)
E-C182/G350	1 (1.03%)	1 (0.81%)	-	-	-	-	-	2 (0.61%)
**E-C183/G350	3 (3.09%)	-	-	-	-	-	-	3 (0.91%)
E-G185/G350	1 (1.03%)	-	-	1 (1.32%)	-	-	1 (1.1%)	2 (0.61%)
E-A188/G350	-	-	-	1 (1.32%)	-	-	1 (1.1%)	1 (0.30%)
E-C188/G350	11 (11.34%)	13 (10.57%)	5 (26.32%)	8 (10.53%)	2 (15.38%)	-	10 (10.99%)	39 (11.82%)
**E-C188/G310/G350	1 (1.03%)	1 (0.81%)	-	-	-	-	-	2 (0.61%)
**E-G189/T256/G350	-	1 (0.81%)	-	-	-	-	-	1 (0.30%)
E-G257/G350	-	1 (0.81%)	-	-	-	-	-	1 (0.30%)
**E-C306/G350	1 (1.03%)	-	-	-	-	-	-	1 (0.30%)
E-C442/G350	1 (1.03%)	-	-	-	-	-	-	1 (0.30%)
**G535/G350	-	1 (0.81%)	-	-	-	-	-	1 (0.30%)
AA variants	**10 (10.31%)**	**14 (11.38%)**	**4 (21.05%)**	**23 (30.27%)**	**6 (46.15%)**	**1 (50.0%)**	**30 (32.97%)**	**58 (17.58%)**
Class AA-a	**4 (4.12%)**	**11 (8.94%)**	**3 (15.79%)**	**12 (15.79%)**	**5 (38.46%)**	**1 (50.0%)**	**18 (19.78%)**	**36 (10.91%)**
AA-a	3 (3.09%)	11 (8.94%)	3 (15.79%)	12 (15.79%)	5 (38.46 %)	1 (50.0%)	18 (19.78%)	35 (10.61%)
**AA-a/C188	1 (1.03%)	-	-	-	-	-	-	1 (0.30%)
Class AA-c	**6 (6.19%)**	**3 (2.44%)**	**1 (5.26%)**	**11 (14.48%)**	**1 (7.69%)**	**-**	**12 (13.19%)**	**22 (6.67%)**
AA-c	6 (6.19%)	3 (2.44%)	1 (5.26%)	9 (11.84%)	1 (7.69%)	-	10 (10.99%)	20 (6.07%)
**AA-c/G185	-	-	-	1 (1.32%)	-	-	1 (1.1%)	1 (0.30%)
AA-c/C271	-	-	-	1 (1.32%)	-	-	1 (1.1%)	1 (0.30%)
Af variants	**-**	**1 (0.81%)**	**-**	**-**	**-**	**-**	**-**	**1 (0.30%)**
Class Af2-a	**-**	**1 (0.81%)**	**-**	**-**	**-**	**-**	-	**1 (0.30%)**
Af2-a/C109/G403	-	1 (0.81%)	-	-	-	-	-	1 (0.30%)

-,Indicate that the variant was not found.

Associations between the five most frequent HPV 16 E6 variants and LSIL, HSIL and cervical carcinoma were assessed (Table 3), using the HPV 16 E-Prototype as a reference. The 5 variants analyzed showed significant association with CC, but only AA-a variant showed significant association with HSIL. The HPV 16 variant that showed the most risk of developing CC was AA-a (OR = 69.01, CI = 7.57-628.96), followed by E-A176/G350 (OR = 39.82, CI = 4.11-386.04), AA-c (OR = 21.16, CI 2.59-172.56), E-G350 (OR = 13.25, CI = 2.02-87.12) and E-C188/G350 (OR = 10.48, CI = 1.39-78.92).

**Table 3:** **The most common HPV 16 E6 variants in women from Southern Mexico and risk of cervical carcinoma**

**Variants**	**Non-IL N (%)**	**LSIL N (%)**	**OR (CI 95%) P**	**HSIL N (%)**	**OR (CI 95%) P**	**SCC N (%)**	**OR (CI 95%) p**	**ADC N (%)**	**OR (CI 95%) P**	**Other epithelial tumor N (%)**	**Cervical carcinoma N (%)**	**OR (CI 95%) P**
Class E-T350^a^	**23 (23.71%)**	**23 (18.7%)**	**-**	**1 (5.26%)**	**-**	**4 (5.26%)**	**-**	**0 (0%)** ^**b**^	**-**	**0 (0.0%)**	**4 (4.40%)**	**-**
E-Prototype	17 (17.53%)	23 (18.7%)	1*	1 (5.26%)	1*	2 (2.63%)	1*	0 (0%)^b^	1*	-	2 (2.2%)	1*
Class E-G350	**64 (65.98%)**	**85 (69.11%)**	**0.97**		**3.73**		**12.66**		**2.42**			**14.12**
			**0.47-1.99**	**14 (73.68%)**	**0.46-30.44**	**49 (64.47%)**	**1.89-84.92**	**7 (53.85%)**	**0.25-**∞	**1 (50.0%)**	**57 (62.65%)**	**2.19-90.97**
			**0.94**		**0.22**		**0.009**		**0.44**			**0.005**
E-G350	39 (40.21%)	56 (45.53%)	1.01		2.58		12.56		1.74			13.25
			0.47-2.17	6 (31.58%)	0.29-23.17	28 (36.84%)	1.84-85.86	3 (23.08%)	0.15-∞	-	31 (34.07%)	2.02-87.12
			0.98		0.39		0.010		0.65			0.007
E-A176/G350	3 (3.09%)	5 (4.07%)	1.49		5.82		24.79		12.62			39.82
			0.31-7.23	1 (5.26%)	0.28-121.37	5 (6.58%)	2.33-263.35	2 (15.38%)	0.79-∞	1 (50.0%)	8 (8.79%)	4.11-386.04
			0.62		0.26		0.008		0.07			0.001
E-C188/G350	11 (11.34%)	13 (10.57%)	1.0		7.91		8.56		3.05			10.48
			0.35-2.84	5 (26.32%)	0.81-77.58	8 (10.53%)	1.07-68.26	2 (15.38%)	0.23-∞	-	10 (10.99%)	1.39-78.92
			0.99		0.08		0.043		0.40			0.023
Class AA-a	**4 (4.12%)**	**11 (8.94%)**	**2.18**		**12.65**		**35.21**		**21.24**			**54.51**
			**0.58-8.17**	**3 (15.79%)**	**1.02-156.20**	**12 (15.79%)**	**3.87-320.39**	**5 (38.46%)**	**1.75-**∞	**1 (50.0%)**	**18 (19.78%)**	**6.36-467.62**
			**0.25**		**0.048**		**0.002**		**0.016**			**0.000**
AA-a	3 (3.09%)	11 (8.94%)	3.08		17.40		43.73		26.69			69.01
			0.73-13.04	3 (15.79%)	1.32-229.60	12 (15.79%)	4.55-420.56	5 (38.46%)	2.10-∞	1 (50.0%)	18 (19.78%)	7.57-628.96
			0.13		0.03		0.001		0.01			0.000
Class AA-c	**6 (6.19%)**	**3 (2.44%)**	**0.44**		**2.79**		**24.46**		**3.01**			**25.28**
			**0.10-2.06**	**1 (5.26%)**	**0.15-52.20**	**11 (14.47%)**	**2.89-207.12**	**1 (7.69%)**	**0.15-**∞	**-**	**12 (13.19%)**	**3.12-204.74**
			**0.30**		**0.49**		**0.003**		**0.47**			**0.002**
AA-c	6 (6.19%)	3 (2.44%)	0.46		2.93		19.97		2.99			21.16
			0.10-2.15	1 (5.26%)	0.16-54.90	9 (11.84%)	2.34-170.25	1 (7.69%)	0.15-∞	-	10 (10.99%)	2.59-172.56
			0.32		0.47		0.006		0.47			0.004
Class Af2-a	**0 (0%)**	**1 (0.81%)**	**-**	**0 (0%)**	**-**	**0 (0%)**	**-**	**0 (0%)**	**-**	**0 (0%)**	**0 (0%)**	**-**

*,Indicate reference category (E-Prototype).

^a^ ,OR was not calculated for E-T350 class because it includes reference category (E-Prototype).

^b^,To calculate OR and CI in ADC an artificial case was created.

OR: Odds ratio adjusted for age.

The novel HPV 16 E6 variants were found in 11 women, 6 from the center of the State of Guerrero, 2 from the coast and 3 from Northern Guerrero. These variants were found in samples taken from 1997 to 2012. The AA-a/C188 HPV 16 variant was found in non-IL cervical sample taken in 1997, AA-c/G185 variant was found in a cervical carcinoma sample taken in 1998, E-A176/G424/G350 was found in a cervical carcinoma sample taken in 2003, E-C183/G350 was found in a non-IL cervical samples taken in 2002 and 2006, E-C306/G350 was found in non-IL a cervical sample taken in 2003, E-C188/G310/G350 was found in a non-IL cervical sample taken in 2004 and an LSIL taken in 2009, E-G535/G350 was found in an LSIL cervical sample taken in 2006 and E-G189/T256/G350 variant was found in an LSIL cervical sample taken in 2008. Six novel variants were found with non-IL, 3 with LSIL and 2 with cervical carcinoma (E-A176/G350/G424 and AA-c/G185). Data from a follow up cytological diagnosis of 5 women with HPV 16 E6 novel variants were collected. Follow up information shows that two with the variant E-C183/G350 evolved from non-IL to LSIL, whereas those with E-C188/G310/G350 and E-G189/T256/G350 maintain LSIL status (Table 4).

**Table 4:** **Novel HPV16 E6 variants, regional distribution, collection year and lesion in cervical epithelium**

**Patient**	**HPV 16 E6 Novel variant**	**Accession number GenBank**	**Nucleotide change**	**Amino acid change (not previously reported)**	**Women residence (City/State region)**	**Diagnosis/Sampling year**	**Follow up Diagnosis/Year**
1	E-A176/G424/G350	KJ465994	G424	-	Atoyac/Coast	Cervical carcinoma 2003	-
2	E-C183/G350	KJ465995	C183	I27T	Chilpancingo/Center	Non-IL 2002	LSIL 2006
3	E-C183/G350	KJ465995	C183	I27T	Chilpancingo/Center	Non-IL 2006	LSIL 2009
4	E-C183/G350	KJ465995	C183	I27T	Chilpancingo/Center	Non-IL 2002	-
5	E-C188/G310/G350	KJ465996	G310	-	Chilpancingo/Center	Non-IL 2004	Non-IL 2005
6	E-C188/G310/G350	KJ465996	G310	-	Chilpancingo/Center	LSIL 2009	LSIL 2012
7	E-G189/T256/G350	KJ465998	G189 and T256	-	Chilpancingo/Center	LSIL 2008	LSIL 2009
8	E-C306/G350	KJ465997	C306	K68T	Acapulco Coast	Non-IL 2003	-
9	E-G535/G350	KJ465999	G535	-	Tepecoacuilco/North	LSIL 2006	-
10	AA-a/C188	KJ465992	C188	-	Iguala/North	Non-IL 1997	-
11	AA-c/G185	KJ465993	G185	-	Juchitan/North	Cervical carcinoma 1998	-

### Sequence Data

The 8 novel variant sequences described in this report have been deposited in GenBank under designated accession numbers KJ465992, KJ465993, KJ465994, KJ465995, KJ465996, KJ465997, KJ465998 and KJ465999.

## Discussion

The objective of this study was to document HPV 16 E6 variants circulating over a period of 16 years in women from Southern Mexico and to analyze its association with cervical carcinoma and precursor lesions. According to sequence analysis, nucleotide polymorphisms were detected and used to investigate the intratypic heterogeneity of HPV 16 in the Southern Mexican population.

It is known that the genomes of HPV 16 variants differ geographically worldwide due to evolution linked to ethnic groups and that the risk for cervical carcinoma seems to be population-dependent [5,6,8,10,26-28]. Mexico is a country with diverse ethnic origins because European immigrants mixed with various indigenous populations, in consequence current population carries HPV variant from various ethnic group [8]. In the present study of 330 women with HPV 16 sampled over a period of 16 years, 27 variants were found; E variants were the most common, followed by AA variants. Studies worldwide have found that E variants are the most prevalent worldwide (94% in Oceania, 84% in Eastern Asia, 83% in North America, 82% in Europe, 78% in Western Asia and 71% in Central and South America) with exception of Africa (36%) [9]. Tornesello, et al. (2011) showed that globally, the most prevalent variant in Central and South America (including Mexico) is E-G350 (43%) followed by AA (30%) and E-Prototype (27%). In North America it is E-Prototype and E-G350 (49% each one) followed by AA (11%). In Europe it is E-G350 (44%) followed by E-Prototype (38%) and AA (6%). In Western Asia it is E-G350 (51%) followed by E-Prototype (25%) and AA (9%). In Eastern Asia it is As (42%) followed by E-Prototype (37%) and E-G350 (9%). In Oceania it is E-Prototype (38%) followed by E-G350 (29%) and As (12%) and in Africa it is Afr1 and Afr2 (62%) followed by E-Prototype (34%). In the present study, the most frequently identified HPV 16 variant was E-G350 (40%), following the E-Prototype (13.03%), E-C188/G350 (11.82%), AA-a (10.61%), AA-c (6.07%) and E-A176/G350 (5.15%). However, unlike other regions, it was found that E-Prototype frequency in Southern Mexico is lower than the rest of the world, while E-G350, considering all its subclasses together, is more frequent than in the rest of the world. The AA variants of HPV 16 were 15-fold more prevalent than E-prototype in cervical carcinoma.

Studies on HPV 16 variants in Mexico have shown that even in the same country its distribution is different depending on the region analyzed. The prevalence of HPV 16 variants, stratified by histological groups, from five geographical regions of Mexico (Central, North-Central, Northeastern, Southeastern and Southern) is presented in Table 5. The E variants are the most prevalent in all geographical regions in women, E-prototype in the Southeastern region and E-G350 in Central and Southern Mexico. AA variants are present in the five regions, but its prevalence is higher in the Northeastern region than in the rest of the country, although it is inhabited mostly by Europeans descendants, Mestizo and very few indigenous ethnic groups [5,8,20,21,23,29]. The State of Guerrero, located in Southern Mexico, is inhabited by Mestizo, Nahuas, Mixtecs, Amuzgos, Tlapanecos and Afro-Mexicans. In this study, which is larger than our previous study [24], we found that HPV 16 E-G350 was the most common variant in all histological grades, although in ADC, the prevalence of E-G350 and AA is close. In all regions of Mexico, the prevalence of AA in ADC tends to increase in comparison to the other histological grades. Moreover, among AA variants, AA-a is more common than AA-c.

**Table 5:** **Published data on HPV 16 variants distribution in cervical epithelium lesions in regions of Mexico**

**Region of Mexico**	**State/City**	**Genome region**	**Samples**	**Histology**	**HPV 16 E**	**HPV 16 E-Prototype**	**HPV 16 E-G350**	**AA**	**AA-a**	**AA-c**	**Reference**
Central	Mexico City	E6, L1	80	SCC	56%	4%	52%	44%	-	-	Berumen, et al., 2001
			6	ADC	0%	0%	0%	100%	-	-	
			20	Non-IL	90%	5%	85%	10%	-	-	
Central	Mexico City	E6	50	SCC	30%	-	-	40%	32%	8%	Lizano, et al., 2006
			11	ADC	55%	-	-	45%	27%	18%	
			23	HSIL	74%	-	-	26%	26%	0%	
			13	LSIL	54%	-	-	46%	46%	0%	
			16	Non-IL	56%	-	-	44%	44%	0%	
North-Central	San Luis Potosí	E6	2	ICC	100%	50%	50%	0%	0%	0%	López-Revilla, et al., 2009
			9	HSIL	89%	33%	56%	11%	11%	0%	
			27	LSIL	96%	85%	11%	4%	4%	0%	
Northeastern	Nuevo León	LCR	112	Undefined	13%	-	-	87%	-	-	Calleja-Macias, et al., 2004
Southeastern	Yucatan	E6	25	IIC	52%	28%	24%	44%	-	-	González-Losa, et al. 2004
			15	LSIL	100%	66%	33%	0%	0%	0%	
Southern	Guerrero	E6	45	ICC	67%	-	-	33%	-	-	Illades-Aguiar, et al., 2010
			10	HSIL	90%	-	-	10%	-	-	
			18	LSIL	67%	-	-	33%	-	-	
			5	Non-IL	60%	-	-	40%	-	-	
Southern	Guerrero	E6	76	SCC	70%	3%	64%	30%	16%	14%	
			13	ADC	54%	-	54%	46%	38%	8%	
			19	HSIL	79%	5%	74%	21%	16%	5%	Present study
			123	LSIL	88%	19%	69%	11%	9%	2%	
			97	Non-IL	90%	18%	66%	10%	4%	6%	

Of the 27 HPV 16 variants found in Southern Mexico in 16 years, 8 of them were new and may be considered to be variants specific to this Mexican region.

Previous data suggests that HPV 16 variants with E6 sequence variation are biologically distinct and may confer different pathological risks for development of squamous intraepithelial lesions and invasive cervical carcinoma. E6 specific sequence variations may modify its linkage to cellular targets changing its ability for p53 degradation, inhibiting keratynocyte differentiation, modifying signal transduction [30-34].

It was proposed that there might be a strong relation between AA variant and cervical carcinoma development [9,16,25,35,36]. Using a comparative analysis, it was found in this study that HPV 16 AA-a detection rate increased according to the severity of the cervical lesion, with a large increase in ADC. The results show that HPV 16 AA-a infection has a strong association with a high risk of CC development compared to E-Prototype. This study reinforces the proposal that HPV 16 AA-a is an oncogenic risk for cervical carcinoma progression in Mexico [5]. Similar behavior was observed in HPV 16 E-A176/G350 variant, the rate increased according severity of the cervical lesion, although frequency was less than for AA-a, and the infection is associated with a risk for development of CC compared with E-Prototype, but less than for AA-a. AA-c, E-G350 and E-C188/G350, also show association with risk for CC compared with E-Prototype, although lower than the aforementioned. The results of this study highlight the importance of identifying HPV 16 variants in the screening of clinical samples and of conducting follow up test for women with the HPV 16 AA-a variant.

According to the HPV variants found in this study, the amino acid substitutions circulating in Southern Mexico are: Q3E, R10G, Q14H, D25N, I27L, I27R I27T, L28V, E29K, E29Q, E29G, I52L, K68T, F69L, H78Y, L83V (the most frequent) and E113D. The amino acid substitutions present in the HPV 16 E6 variants in descending order of association with CC were: Q14R/H78Y/L83V (AA-a variant), D25N/L83V (E-A176/G350 variant), Q14R/I27/H78Y/L83V (AA-c variant), L83V (E-G350 variant) and E29Q/L83V (E-C188/G350 variant). Q14R/H78Y/L83V amino acid changes gender AA variants with increased oncogenic potential and more efficient evasion of the host’s immune surveillance [25,37,38]. L83V amino acid change display more efficient degradation of Bax and binding to E6AP, induces ubiquitination and degradation of p53, NFX1-91 and PDZ proteins [14,33]. D25N, I27R and E29Q amino acid changes affect E6 T cell epitope [33]. Two of the 8 novel variants found in this study had amino acid changes not previously reported: I27T in E-C183/G350 variant and K68T in E-C306/G350 variant. I27T amino acid change is located at the N-terminal domain of the E6 oncoprotein; K68T is located between zinc fingers of E6. The E-C183/G350 variant was found in women with non-IL who progressed to LSIL. The finding of this new variant could be potentially important.

It was not possible to analyze the association of novel E6 variants for the risk of developing cervical carcinoma because of the low number of positive samples; however, by having nucleotide changes may also be associated with the development of cervical carcinoma.

## Conclusions

Current findings show that in 16 years, at least 27 HPV 16 E6 variants were present in Southern Mexico and 8 novel variants were found, which may be considered to be variants specific to this Mexican region. The variants more frequently found in women with cervical carcinoma are E-G350, AA-a, AA-c, E-C188/G350 and E-A176/G350. All of them are associated with the development of cervical carcinoma, however, AA-a showed the highest association. This study reinforces the proposal that HPV 16 AA-a is an oncogenic risk for cervical carcinoma progression in Mexico. This represents the largest study carried out in Mexico analyzing all classes of European and non-European variants in the whole spectrum of disease, from intraepithelial lesion-free cytology to cervical carcinoma including squamous cell carcinoma and adenocarcinoma. Further studies are needed to clarify the pathogenicity of HPV 16 E6 variants.

## Methods

### Samples

The database and biobank, with 7,480 cervical DNA samples collected from 1997 to 2012, of the Molecular Biomedicine and Cytopathology Laboratories at the School of Chemistry and Biology of the Autonomous University of Guerrero in Chilpancingo Guerrero, Mexico, was searched for all cervical DNA samples with HPV 16 and 330 were found in appropriate conditions for analysis. The samples were studied to investigate circulating HPV 16 variants in Southern Mexico and to do a comparative analysis between these variants and the different grades of cervical lesion.

Cervical samples came from women who were residents of State of Guerrero, seeking cytological screening or for other gynecological complaints, which attended public health centers of Acapulco, Chilpancingo, and Iguala, the three biggest cities in this state of Southern Mexico. Based on the diagnosis, samples were divided into: (1) no intraepithelial lesion (non-IL) (n = 97), (2) low-grade squamous intraepithelial lesion (LSIL) (n = 123), high-grade squamous intraepithelial lesion (HSIL) (n = 19) and (4) cervical carcinoma (CC) (n = 91). Non-IL and LSIL samples have cytological diagnosis; HSIL and CC samples have histological diagnosis. Cytological diagnosis was done according to the Bethesda System [39] and histological diagnosis according to the classification system of the International Federation of Gynecology and Obstetrics (FIGO) [40].

This study was approved by the Bioethical Committee the Autonomous University of Guerrero. Informed consent was obtained from women participants.

HPV DNA was detected and identified by three methods depending on the year of in which the sample was taken and analyzed: (1) from 1997 to 2010, HPV detection was done by the MY09/11 system and typing by restriction fragment length polymorphism (RFLPs); (2) from 2005 to 2010, detection was done by general GP5+/6+ PCR system and typing by sequencing analysis [24] when samples analyzed with MY09/11 PCR were negative; (3) from 2010 to 2012 HPV was detected and typed with INNO Lipa genotyping Extra (Innogenetics) [41].

### HPV variants

The HPV 16 E6 gene was amplified using type-specific primers E6-F048 (5′GAACCGAAACCGGTTAGTAT3′) and E6-R622 (5′CAGTTGTCTCTGGTTGCAAA3′) that amplify a 575-bp region [19]. PCR amplification was carried out in a 50 μl reaction containing 1 μM of each primer, 4 mM of MgCl_2_, and 1.25 U of Platinum Taq DNA Polymerase (Invitrogen). The DNA amplification was done in a DNA Eppendorf AG 22331 Hamburg [24] as follows: initial denaturation at 95°C for 10 min, followed by 40 cycles of 95°C for 45 s, 57°C for 30 s, 72°C for 1.15 min, and a final extension al 72°C for 10 min.

PCR products were purified with 75% isopropanol (2–34 protocol of user manual of Applied Biosystems) and ZR DNA Sequencing Clean-up Kit™ (ZYMO RESEARCH). These were sequenced using Big Dye Terminator Chemistry v3.1 Ready Reaction Kit (Applied Biosystems, Foster City, CA) in an automated sequencer DNA ABI Prism 310 Genetic Analyzer (Applied Biosystems, Foster City, CA) using primers previously described [19]. Sequences were analyzed with EMBOSS Stretcher of the European Institute of Bioinformatics, LALING GENESTREAM network server (http://www.ebi.ac.uk/Tools/psa/emboss_stretcher/nucleotide.html and http://embnet.vital-it.ch/software/LALIGN_form.html) and the Finch TV program respectively. Sequences were aligned with reference sequence (HPV 16R) [8]. Using the E6 sequence, HPV 16 variants were classified into lineages with their respective sublineages [12]. The sublineages were stratified in classes and subclasses [13]. When new polymorphisms were found, independent PCRs were carried out under the described conditions. The products obtained were sequenced on both strands to exclude PCR artifacts and to validate the polymorphism found and accept them as new variants.

### Phylogenetic analysis

HPV 16 E6 sequences were compared by multiple sequence alignments using the CLUSTAL W method [42]. A phylogenetic tree was constructed by neighbor-joining analysis executed by MEGA 5.2 program [43].

### Statistical analysis

The Chi squared test was used to compare HPV 16 variant frequencies and cervical lesion grade. Differences were considered to be statistically significant when *p* values were less than 0.05. Age-adjusted odds ratios and 95% confidence intervals were used to estimate associations. Data analysis and statistics were done using IBM SPSS Statistics V.22.0 and STATA V.11 softwares.

## References

[1] Zur Hausen H (2002). Papillomaviruses and cancer: from basic studies to clinical application. Nat Rev Cancer.

[2] Guan P, Howell-Jones R, Li N, Bruni L, de Sanjose S, Franceschi S, Clifford GM (2012). Human papillomavirus types in 115,789 HPV-positive women: a meta-analysis from cervical infection to cancer. Int J Cancer.

[3] Peitsaro P, Johansson B, Syrjänen S (2002). Integrated human papillomavirus type 16 is frequently found in cervical cancer precursors as demonstrated by a novel quantitative real-time PCR technique. J Clin Microbiol.

[4] Gheit T, Cornet I, Clifford GM, Iftner T, Munk C, Tommasino M, Kjaer SK (2011). Risks for persistence and progression by human papillomavirus type 16 variant lineages among a population-based sample of Danish women. Cancer Epidemiol Biomarkers Prev.

[5] Berumen J, Ordoñez RM, Lazcano E, Salmerón J, Galván SC, Estrada RA, Yunes E, García-Carranca A, Gonzalez-Lira G, Madrigal-de la Campa A (2001). Asian-American variants of human papillomavirus 16 and risk for cervical cancer: a case–control study. J Natl Cancer Inst.

[6] Zuna RE, Moore WE, Shanesmith RP, Dunn ST, Wang SS, Schiffman M, Blakey GI, Teel T (2009). Association of HPV16 E6 variants with diagnostic severity in cervical cytology samples of 354 women in a US population. Int J Cancer.

[7] Schiffman M, Rodríguez AC, Chen Z, Wacholder S, Herrero R, Hildesheim A, Desalle R, Befano B, Yu K, Safaeian M, Sherman ME, Morales J, Guillen D, Alfaro M, Hutchinson M, Solomon D, Castle PE, Burk RD (2010). A Population-Based Prospective Study of Carcinogenic Human Papillomavirus Variant Lineages, Viral Persistence, and Cervical Neoplasia. Cancer Res.

[8] Calleja-Macías IE, Kalantari M, Huh J, Ortiz-López R, Rojas-Martínez A, Gonzalez-Guerrero JF, Williamson AL, Hagmar B, Wiley DJ, Villarreal L, Bernard HU, Barrera-Saldaña HA (2004). Genomic diversity of human papilomavirus- 16, 18, 31 and 35 isolates in a Mexican population and relationship to European, African, and Native American variants. Virol.

[9] Tornesello ML, Losito S, Benincasa G, Fulciniti F, Botti G, Greggi S, Buonaguro L, Buonaguro FM (2011). Human papillomavirus (HPV) genotypes and HPV16 variants and risk of adenocarcinoma and squamous cell carcinoma of the cervix. Gynecol Oncol.

[10] Cornet I, Gheit T, Lannacone MR, Vignat J, Sylla BS, Del Mistro A, Franceschi S, Tommasino M, Clifford GM, IARC HPV, Variant Study Group (2013). HPV16 genetic variation and the development of cervical cancer worldwide. Br J Cancer.

[11] Chen Z, Schiffman M, Herrero R, De Salle R, Anastos K, Segondy M, Sahasrabuddhe VV, Gravitt PE, Hsing AW, Burk RD (2011). Evolution and Taxonomic Classification of Human Papillomavirus 16 (HPV16)-Related Variant Genomes: HPV31, HPV33, HPV35, HPV52, HPV58 and HPV67. Plos One.

[12] Cornet I, Gheit T, Franceschi S, Vignat J, Burk RD, Sylla BS, Tommasino M, Clifford GM, IARC HPV Variant Study Group (2012). Human Papillomavirus Type 16 Genetic Variants: Phylogeny and Classification Based on E6 and LCR. J Virol.

[13] Huertas-Salgado A, Martín-Gámez DC, Moreno P, Murillo R, Bravo MM, Villa L, Molano M (2011). E6 molecular variants of human papillomavirus (HPV) type 16: An updated and unified criterion for clustering and nomenclature. Virol.

[14] Lichtig H, Algrisi M, Botzer LE, Abadi T, Verbitzky Y, Jackman A, Tommasino M, Zehbe I, Sherman L (2006). HPV16 E6 natural variants exhibit different activities in functional assays relevant to the carcinogenic potential of E6. Virol.

[15] Zehbe I, Richard C, Decarlo CA, Shai A, Lambert PF, Lichtig H, Tommasino M, Sherman L (2009). Human papillomavirus 16 E6 variants differ in their dysregulation of humankeratinocyte differentiation and apoptosis. Virol.

[16] Zehbe I, Lichtig H, Westerback A, Lambert PF, Tommasino M, Sherman L (2011). Rare human papillomavirus 16 E6 variants reveal significant oncogenic potential. Mol Cancer 2011.

[17] Smith B, Chen Z, Reimers L, Doorslaer K, Schiffman M, DeSalle R, Herrero R, Yu K, Wacholder S, Wang T, Burk RD (2011). Sequence Imputation of HPV16 Genomes for Genetic Association Studies. PLoS ONE.

[18] Jang M, Rhee JE, Jang DH, Kim SS (2011). Gene Expression Profiles are Altered in Human Papillomavirus-16 E6 D25E-Expressing Cell Lines. Virol J.

[19] Casas L, Galván SC, Ordoñez RM, López N, Guido M, Berumen J (1999). Asian-american variants of human papillomavirus type 16 have extensive mutations in the E2 gene and are highly amplified in cervical carcinomas. Int J Cancer.

[20] Lizano M, Berumen J, García-Carranca A (2009). HPV-related Carcinogenesis: Basic Concepts, Viral Types and Variants. Arch Med Res.

[21] López-Revilla R, Pineda MA, Ortíz-Valdez J, Sánchez-Garza M, Riego L: Human papillomavirus type 16 variants in cervical intraepithelial neoplasia and invasive carcinoma in San Luis Potosi City, Mexico. Infectious Agents and Cancer 2009, **4**(3): http://www.infectagentscancer.com/content/4/1/3. 10.1186/1750-9378-4-3 PMC2653482 19216802

[22] Bradley CJ, Given CW, Roberts C (2004). Health Care Disparities and Cervical Cancer. Am J Public Health.

[23] Secretaría de Salud. Dirección General de Información en Salud. Mexico, 2006. Available from URL: http://www.dgis.salud.gob.mx/descargas/xls/m_016.xls. Accessed March 27, 2009.

[24] Illades-Aguiar B, Alarcón-Romero LC, Antonio-Véjar V, Zamudio-López N, Sales-Linares N, Flores-Alfaro E, Fernández-Tilapa G, Vences-Velázquez A, Muñoz-Valle JF, Leyva-Vázquez MA (2010). Prevalence and distribution of human papillomavirus types in cervical cancer, squamous intraepithelial lesions, and with no intraepithelial lesions in women from Southern Mexico. Gynecol Oncol.

[25] Richard C, Lanner C, Naryzhny SN, Sherman L, Lee H, Lambert PF, Zehbe I (2010). The immortalizing and transforming ability of two common human papillomavirus 16 E6 variants with different prevalences in cervical cancer. Oncogene.

[26] Yamada T, Manos MM, Peto J, Greer C, Muñoz N, Bosh FX, Wheeler M (1997). Human papillomavirus type 16 sequence variation in cervical cancers: a wordlwide perspective. J Virol.

[27] Villa LL, Sichero L, Rahal P, Caballero O, Ferenczy A, Rohan T, Franco EL (2000). Molecular variants of human papillomavirus types 16 and 18 preferentially associated with cervical neoplásica. J Gen Virol.

[28] Tornesello ML, Duraturo ML, Salatiello I, Buonaguro L, Losito S, Botti G, Stellato G, Greggi S, Piccoli R, Pilotti S, Stefanon B, De Palo G, Franceschi S, Buobaguro FM (2004). Analysis of human papillomavirus type-16 variants in Italian women with cervical intraepithelial neoplasia and cervical cancer. J Med Virol.

[29] González-Losa MDR, Laviada Mier y Teran MA, Puerto-Solís M, García-Carrancá A (2004). Molecular variants of HPV type 16 E6 among Mexican women with LSIL and invasive cancer. J Clin Virol.

[30] Zehbe I, Voglino G, Wilander E, Delius H, Marongiu A, Edler L, Klimek F, Andersson S, Tommasino M (2001). p53 codon 72 polymorphism and various human papillomavirus 16 E6 genotypes are risk factors for cervical cancer development. Cancer Res.

[31] Xi LF, Koutsky LA, Hildesheim A, Galloway DA, Wheeler CM, Winer RL, Ho J, Kiviat NB (2007). Risk for High-Grade Cervical Intraepithelial Neoplasia Associated with Variants of Human Papillomavirus Types 16 and 18. Cancer Epidemiol Biomarkers Prev.

[32] Chopjitt P, Ekalaksananan T, Pientong C, Kongyingyoes B, Kleebkaow P, Charoensri N (2009). Prevalence of human papillomavirustype16 and its variants in abnormal squamous cervical cells in Northeast Thailand. Int J Infect Dis.

[33] Pillai MR, Hariharan R, Babu JM, Lakshmi S, Chiplunkar SV, Patkar M, Tongaonkar H, Dinshaw K, Jayshree RS, Reddy BK, Siddiqui M, Roychoudury S, Saha B, Abraham P, Gnanamony M, Peedicayil A, Subhashini J, Ram TS, Dey B, Sharma C, Jain SK, Singh N (2009). Molecular variants of HPV-16 associated with cervical cancer in Indian population. Int J Cancer.

[34] Wise-Draper TM, Wells SL (2008). Papillomavirus E6 and E7 proteins and their cellular targets. Front Biosci.

[35] Junes-Gill K, Sichero L, Maciag PC, Mello W, Noronha V, Villa LL (2008). Human papillomavirus type 16 variants in cervical cancer from an admixtured population in Brazil. J Med Virol.

[36] Quint KD, de Koning MN, van Doorn LJ, Quint WG, Pirog EC (2010). HPV genotyping and HPV16 variant analysis in glandular and squamous neoplastic lesions of the uterine cervix. Gynecol Oncol.

[37] Zehbe I, Mytilineos J, Wikström I, Henriksen R, Edler L, Tommasino M (2003). Association between human papillomavirus 16 E6 variants and human leukocyte antigen class I polymorphism in cervical cancer of swedish women. Hum Immunol.

[38] Sichero L, Sobrinho JS, Villa LL (2012). Oncogenic potential diverge among human papillomavirus type 16 natural variants. Virol.

[39] Solomon D, Davey D, Kurman R, Moriarty A, O'Connor D, Prey M, Raab S, ShermanM W, Wright TJ, Young N (2002). The 2001 Bethesda System: terminology for reporting results of cervical cytology. JAMA.

[40] Benedet JL (2000). FIGO staging classifications and clinical practice guidelines of gynaecologic cancers. FIGO Committee on Gynecologic Oncology. Int J Gynecol Obstet.

[41] Kleter B, van Doorn LJ, Schrauwen L, Molijn A, Sastrowijoto S, ter Schegget J, Lindeman J, ter Harmsel B, Burger M, Quint W (1999). Development and clinical evaluation of a highly sensitive PCR-reverse hybridization line probe assay for detection and identification of anogenital human papillomavirus. J Clin Microbiol.

[42] Thompson JD, Gibson TJ, Plewniak F, Jeanmougin F, Higgins DG (1997). The CLUSTAL_X windows interface: flexible strategies for multiple sequence alignment aided by quality analysis tools. Nucleic Acids Res 1997.

[43] Tamura K, Peterson D, Peterson N, Stecher G, Nei M, Kumar S (2011). MEGA5: Molecular Evolutionary Genetics Analysis using Maximum Likelihood, Evolutionary Distance, and Maximum Parsimony Methods. Mol Biol Evol.

